# The global health community at international climate change negotiations

**DOI:** 10.1136/bmjgh-2024-015292

**Published:** 2024-04-18

**Authors:** Kim Robin van Daalen, Nanine Wyma, Johanna Schauer-Berg, Iris M Blom, Juliette Mattijsen, Razan Othman, Mohamed Eissa, Robbie M Parks, Arthur Wyns, Ahmed Taha Aboushady, Muha Hassan, Tarek Ezzine, Salman Khan, Menna-Allah Elsayed Zayed, Sarah Neggazi, Lujain Alqodmani, Rachel Lowe

**Affiliations:** 1 Barcelona Supercomputing Center (BSC), Barcelona, Spain; 2 British Heart Foundation Cardiovascular Epidemiology Unit, Department of Public Health and Primary Care, University of Cambridge, Cambridge, UK; 3 Heart and Lung Research Institute, University of Cambridge, Cambridge, UK; 4 African Unit for Transdisciplinary Health Research (AUTHeR), North-West University, Potchefstroom, South Africa; 5 Physicians Association for Nutrition South Africa, Pinelands, South Africa; 6 Institute of General Practice, Family Medicine and Preventive Medicine, Paracelsus Medical University (PMU), Salzburg, Austria; 7 Centre on Climate Change and Planetary Health, London School of Hygiene and Tropical Medicine, London, UK; 8 Julius Center for Health Sciences and Primary Health, University Medical Centre Utrecht, Utrecht, Netherlands; 9 Department of Public Health, Erasmus Medical Center, Rotterdam, Zuid-Holland, Netherlands; 10 The National Ribat University, Khartoum, Khartoum, Sudan; 11 Faculty of Medicine, Alexandria University, Alexandria, Egypt; 12 Department of Environmental Health Sciences, Mailman School of Public Health, Columbia University, New York, New York, USA; 13 Melbourne Climate Futures, The University of Melbourne, Melbourne, Victoria, Australia; 14 Brigham and Women’s Hospital, Harvard Medical School, Boston, Massachusetts, USA; 15 Wye Valley NHS Trust, Hereford, Herefordshire, UK; 16 Faculty of Medicine of Tunis, University of Tunis El Manar, Tunis, Tunisia; 17 Grant Government Medical College and Sir J J Group of Hospitals, Mumbai, Maharashtra, India; 18 International Federation of Medical Students' Associations (IFMSA), Copenhagen, Denmark; 19 Faculty of Pharmacy, Alexandria University, Alexandria, Egypt; 20 International Pharmaceutical Federation, The Hague, South Holland, Netherlands; 21 Faculty of Pharmacy, University of Algiers 1, Alger, Algeria; 22 International Pharmaceutical Students’ Federation, the Hague, Netherlands; 23 World Medical Association, Ferney-Voltaire, France; 24 Centre on Climate Change & Planetary Health and Centre for Mathematical Modelling of Infectious Diseases, London School of Hygiene & Tropical Medicine, London, UK; 25 Catalan Institution for Research and Advanced Studies (ICREA), Barcelona, Spain

**Keywords:** Global Health, Decision Making, Environmental health, Public Health

**SUMMARY BOX**

- Health played a central role in the recent Conference of Parties 28 (COP28): witnessing the first official ‘Health Day’, the first COP climate-health ministerial, endorsements of a declaration on climate change and health by 149 countries, the highest number of climate and health-related side-events, and funding commitments of US$1 billion dedicated to climate and health.
- In this first-ever quantitative analysis of the health community’s attendance at UN climate conferences between 1995 and 2023, we show a steady increase in absolute attendance of health actors, with the highest attendance of health actors at COP28 (n=1612) compared with the lowest attendance at COP1 (n=17). Yet, the percentage of health delegates remained largely constant over time in relation to the overall number of attendees.
- Although a small number of Ministers of Health attended individual COPs between 1995 and 2022, COP28 was attended by approximately the same number of Health Ministers (n=52) as in all previous COPs combined (n=53).
- While parties and representatives of the UN and its Specialised Bodies increasingly embrace the health narrative, crucial climate change commitments continue to lag.
- Without fundamental social change, without phasing out fossil fuels, and without climate justice, the health narrative for climate change cannot bring what it promises: health for all.

With 2023 shattering climate records across the world following decades of unprecedented warming,^1^ the United Nations Framework Convention on Climate Change Conference of Parties 28 (UNFCCC COP28) in Dubai was the first UN climate change conference to feature an official ‘Health Day’ and witnessed the largest-ever turnout of the global health community. The threat of climate change to human well-being and planetary health^2 3^ has previously received little attention at the annual COPs, despite ever-growing scientific evidence warning of the increasing health dangers.

## Climate change is here and it kills

The widespread negative health impacts of climate change are indisputable, ranging from the (re-)emergence, and increased spread of infectious diseases and increasing non-communicable diseases to escalating exposure to extreme events and climatic shocks undermining the environmental, social, physical and psychosocial determinants of health.^2 3^ These climate-related health impacts are not equally experienced. Structural inequalities exacerbate the vulnerability of specific population groups—such as low-income communities,^4 5^ migrants and displaced people,^6 7^ ethnic minoritised and Indigenous peoples,^4^ people with existing health conditions, as well as sexual and gender minoritised people,^8 9^ children^10^ and women going through pregnancy and childbirth.^11^ Populations most impacted tend to be those least responsible for greenhouse gas (GHG) emissions and those less likely to be prioritised in climate change policies.^12 13^ With negative health trends expected to intensify under all emission scenarios, limiting average global warming to 1.5°C averts further detrimental health impacts and simultaneously delivers health cobenefits, including cleaner air, active transport and healthier diets.^3^

## Health offers climate change a human face

Different ways of framing societal issues can change how priorities are discussed and addressed. Several studies indicate that presenting a positive vision of a healthier, more sustainable common future (ie, using a ‘health’ framing) may increase social and political support for climate action across societal groups and political boundaries.^14 15^ With health being central to many broad-scale regulations and legislations (eg, air quality or food/water standards), health institutions can also build on their institutional capacity to engage directly and meaningfully in climate change policy-making.^15^ Illustratively, in 2009, the US Environmental Protection Agency was able to regulate several GHG gases under the Clean Air Act—based on their authority to protect public health—while environmental legislation to reduce emissions earlier stalled in Congress.^16^

## The health community as agents of change

The global health community views climate change as an important and growing cause of health harms^17^ and increasingly engages with efforts in support of stronger climate action to protect patients, communities and the planet; initiating action to reduce GHG emissions in their professional work, supporting and contributing to building more climate-resilient, sustainable and low-carbon health systems, producing scientific evidence on the links between climate and health, implementing public health measures to prevent and reduce the severity of climate-related health risks, mobilising non-violent protests and spearheading various advocacy efforts.^15^ Health professionals are among the most trusted professions around the world,^18 19^ tending to have close relationships with local communities. Therefore, they are uniquely positioned to advocate for just, health-responsive climate action.

The global health community has actively taken part in UN climate change conferences for over a decade. Yet, health activities have largely been limited to siloed side-events and advocacy efforts, with limited influence on the formal negotiations. Recently, however, health has taken a more pivotal role in proceedings; at COP23, the WHO was tasked for the first time to deliver a special climate and health report for COP24^20^; and during COP26 a dedicated health programme was developed providing a platform for countries to commit to building climate-resilient, sustainable, low-carbon health systems. As of September 2022, 91% of the Nationally Determined Contributions (NDCs) under the Paris Agreement have incorporated health goals and targets.^21^ The most recent COP28 convened the first-ever climate-health ministerial and saw the endorsement of a political declaration on climate and health by 149 countries, potentially fostering further integration of health within the UNFCCC. Furthermore, COP28 hosted the highest number of climate and health-related events (over 200), saw funding commitments of over US$1 billion dedicated to climate and health and US$778.2 million to neglected tropical diseases (NTDs), and motivated more countries to join the Alliance for Transformative Action on Climate and Health (ATACH), a WHO-hosted multinational network dedicated to building climate-resilient, low-carbon healthcare systems.

## The health community at international climate change negotiations: in numbers

While the largest-ever turnout of the global health community at COP28 was widely celebrated, there has been no previous quantitative analysis of the health community’s attendance since the first 1995 COP in Berlin. Here, we analysed health actors’ attendance among representatives of Parties, Observer States and Observer Organisations. These data may further support the assessment of the influence of health and the health community in international climate change negotiations. A broad definition of ‘health community’ or ‘health actor’ was applied, including any person who provides healthcare or treatment, represents a government on matters related to health, works for an organisation or institution primarily focused on human health, or works for organisations representing patients or people with disabilities. The detailed methodology, limitations and data can be found in the online supplemental materials.

10.1136/bmjgh-2024-015292.supp1 Supplementary data

**Figure 1:**
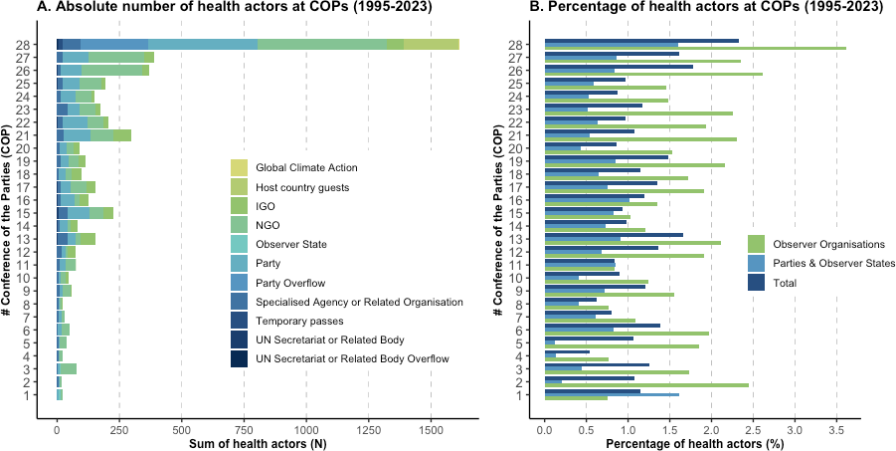
Health actors' attendance at United Nations Framework Convention on Climate Change (UNFCCC)’s COP28 (1995–2023). (A) The absolute number of health actors attending COP1-28. (B) Percentage of health actors attending COP of total participation, grouped by Parties and Observer States and Observer Organisations (representatives of UN Secretariat and Related Bodies, UN Specialised Agencies or Related Organisations, Intergovernmental Organisations [IGOs], Non-Governmental Organisations [NGOs] and for COP28 representatives of Global Climate Action, Host Country Guests, and Temporary Passes). Note, Parties are those that have ratified the Convention and fully engage in negotiations. Observer States are those that have not yet completed their ratification of the Convention, and, therefore, do not yet have the right to vote on decisions. Observer Organisations do not have the right to vote on decisions and have more limited access to the convening (eg, they do have access to the plenary sessions, but not to smaller Party discussions). See the online supplemental material for further details on the methodology, including limitations.

**Figure 2:**
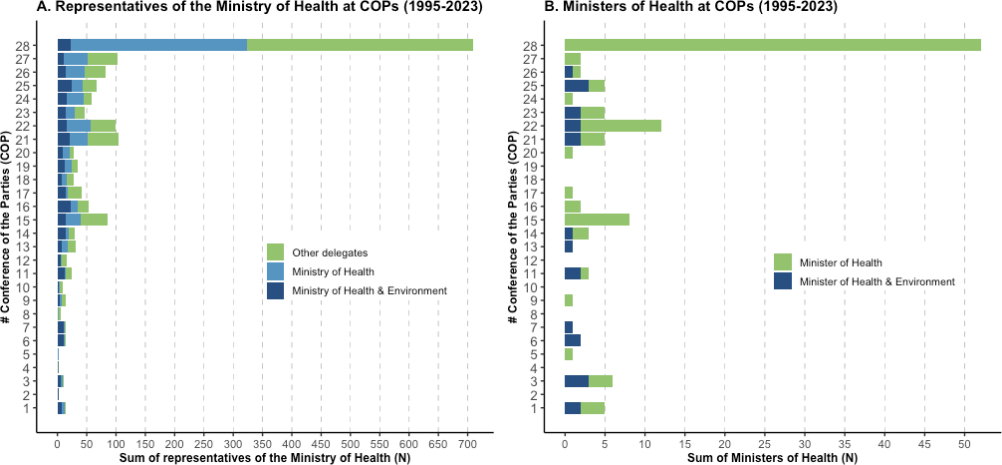
Party and Observer State representatives associated with the Ministry of Health at COPs (1995–2023). (A) Representatives of the Ministry of Health, or Ministry of Health & Environment. (B) Ministers of Health or Ministers of Health & Environment. This figure focuses on representatives of Party and Observer State delegations, excluding representatives from Observer Organisations. See the online supplemental material for further details on the methodology, including limitations.

**Figure 3:**
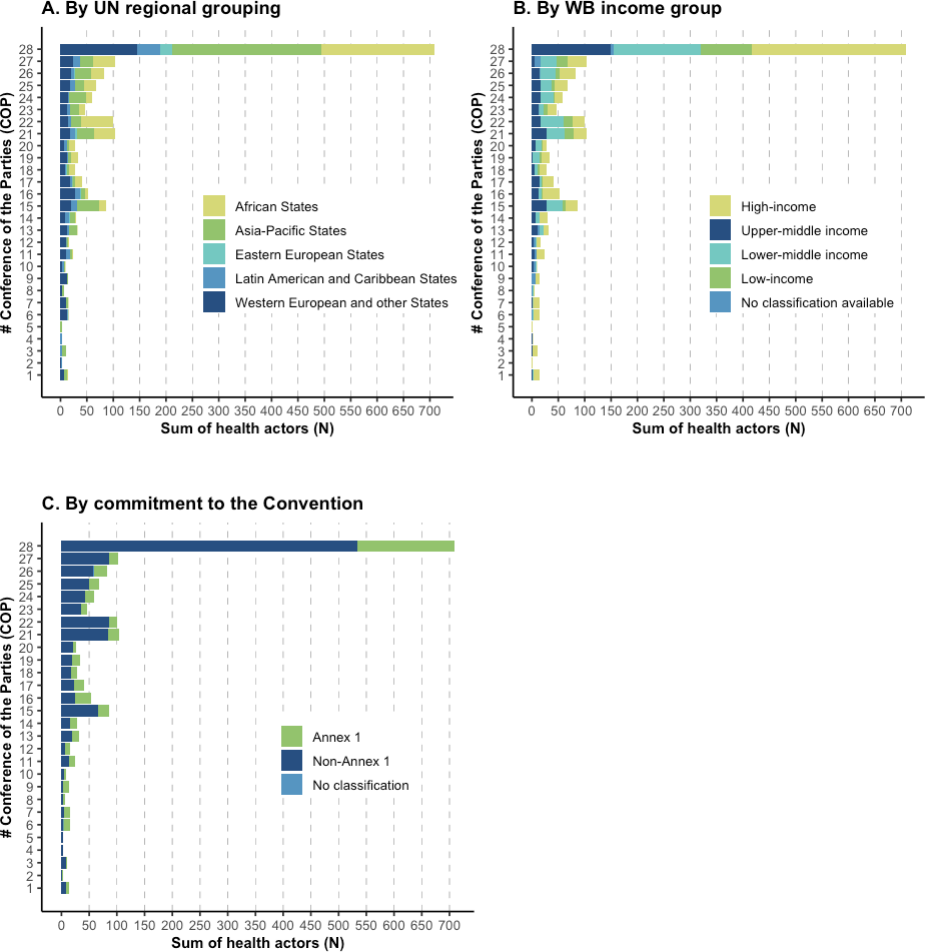
Party and Observer State health actors’ attendance at United Nations Framework Convention on Climate Change (UNFCCC) COP 1–28 (1995–2023) by United Nations (UN) regional grouping and World Bank (WB) country income grouping. This figure excludes representatives from Observer Organisations as no information for their country is provided in UNFCCC documentation. (A) UN regional grouping. Note, health actors’ attendance from Western European and other states has been particularly driven by the attendance of Belgium delegates as part of the combined Ministry of Health and Environment (ie, Santé publique, Sécurité de la chaîne alimentaire et Environnement). (B) World Bank (WB) country income grouping. Parties with no classification available include formerly existing countries (eg, Yugoslavia), country groups (eg, European Union) and those pending release of national account statistics (eg, Venezuela). See the online supplemental appendix for further details on the methodology, including limitations. (C) Commitment to the Convention. Those with no classification include former countries (Yugoslavia, COP6) and Palestine (COP26).

A steady increase in absolute attendance of health actors at UN climate conferences between 1995 and 2023 was observed (figure 1A), with the highest attendance of health actors at COP28 (n=1612), compared with the lowest attendance at COP1 (n=17). However, this increase was correlated with a general increase in the number of COP participants over time (online supplemental table 4 1; online supplemental figure 1), with peaks in attendance at key climate change diplomacy moments such as COP15 (2009; Copenhagen Accord), COP21 (2015; Paris Agreement) and COP28 (2023; Global Stocktake). The percentage of health delegates in relation to the overall number of attendees remained largely constant over time (figure 1B; online supplemental table 4). Health actors were predominantly present as observers (Non-Governmental Organisations (NGOs), Intergovernmental Organisations (IGOs) and UN Specialised Agencies or Related Organisations; figure 1A,B). Participation of government representatives from Ministries of Health has likewise steadily increased in absolute numbers (figure 2A), although some were representatives from combined Health and Environment Ministries (eg, Belgium). Furthermore, while a small number of Ministers of Health attended COP1-27, COP28 was attended by approximately the same number of Health Ministers (n=52) as in all previous COPs combined (n=53) (figure 2B). This may be at least partially explained by additional funding and travel support provided by the COP28 Presidency to 29 Ministers of Health from low and middle-income countries (LMICs), and the inclusion of health in official UNFCCC programming. In absolute numbers, most Party and Observer State health actors were from the Asia-Pacific states (n=585) and African states (n=504; figure 3A), LMICs (n=1007; figure 3B), or non-Annex 1 countries (n=1239; figure 3C) over 1995–2023, representing a major part of those most impacted by climate change. Note, due to a lack of summary data, we could not extend these subgroup analyses meaningfully beyond absolute numbers (see limitations in the online supplemental material for further details).

## The largest-ever turnout of the global health community at COP

The rising number of health actors present at UN Climate Conferences cannot be called a success in and of itself; our analyses show limited relative growth of health actors present. Indeed, overall participation has grown considerably, from around 2000 participants during COP1 to 80 000 participants during COP28. Not only did health actors’ attendance increase over the past years, so did the attendance from health-harming sectors, including 2500 fossil fuel representatives at COP28—a fivefold increase since COP26.^22^ This is happening against a backdrop of oil and gas companies continuing to attract investment, expand infrastructure and report record-breaking profits.^3 23^ Simultaneously, air pollution from fossil fuel burning results in millions of global deaths yearly while the planet approaches the critical 1.5°C planetary boundary.^3^ Notably, our dataset (1995–2023) identified 87 participants with health functions (eg, Health & Safety Officers) in organisations involved in fundamentally health-harming practices such as the exploration, production, refining, distribution, marketing or import/export of oil, coal, petroleum or gas. This suggests that not all health actors present at COP represent the interests of public health.

As a part of the overall rapid increase in the health community’s mobilisation on climate change, the increased health actors’ attendance at COPs might have brought increased attention to the health argument for climate action within UN spaces and international negotiations. The marked increase in the number of Health Ministers and senior representatives from Ministries of Health at COP28 might arguably have strengthened recognition of the role of health in international climate negotiations and might have enabled stronger collaboration between climate and health policy-makers within national delegations and beyond. Continued capacity development for, and representation of, Ministries of Health to meaningfully engage in future UN climate conferences would contribute to sustained and strengthened recognition of health as a pillar for climate change policies.

## Health success at COP28 is accompanied by discomfort

The health perspective offers a compelling and urgent case for ambitious climate action, underscoring the vital role of the health community in driving climate action. When climate policy is aligned with health objectives, the narrative of climate action transforms into a tale of enhanced health and well-being, promoting equity for all. Health renders the effects of climate policies palpable in people’s everyday lives.

Simultaneously, the COP28 health successes also come with considerable discomfort. While the health narrative is increasingly embraced, crucial climate change commitments continue to lag. Consensus-based decisions have failed to reflect Parties’ commitment to the imperative phase-out of fossil fuels, with combined pledges in NDCs putting the world on track for around 2.5°C of warming (exceeding climate tipping points)^24^ and loss and damage funds remaining insufficient to protect most at-risk communities. The question lingers whether health is being used to increase the acceptability of initiatives or organisations that minimally advance climate action (ie, the so-called ‘healthwashing’). Specifically, within the COP28 context, does the current focus on health and celebration of the increased health community presence distract from the fact that climate change commitments remain inadequate to avert imminent climate breakdown?^25^

Furthermore, as differential exposure, sensitivity and adaptive capacity (all impacting vulnerability) result in uneven distributions of climate-related health impacts, often reflecting sociodemographic inequities and marginalisation, a focus on equity within the climate-health realm is imperative to establishing a meaningful, environmentally just transition.^4 26 27^ This includes ensuring the inclusion of diverse voices, perspectives, expertise and lived experiences within climate change negotiations, as well as the climate health community—particularly of those living in regions most affected.^28^ While there is a relatively larger presence of Party health actor representatives from the Global South (with most Party health actors’ representing African or Asia-Pacific states in absolute numbers), they also represent most Parties to the Convention and most of the world’s population. There remains a significant need to increase the Global South participation of health actor representatives, including those representing non-Party delegates such as NGOs.^29 30^ Importantly, representation alone does not necessarily equate to more inclusive decision-making or equal access to power, speaking engagements and informal/formal processes. Prioritising inclusive advocacy efforts that centre equity and address structural barriers to meaningful participation would enable just and transformative decision-making, involving further reductions in global GHG emissions and supporting those most affected by climate change.^31^

## Conclusion

In this quantitative analysis of the health community’s attendance at UN climate conferences between 1995 and 2023, we show a steady increase in absolute attendance of health actors, but limited relative growth compared with overall COP participation. These new indicators may support the assessment of the engagement of health and health actors in international climate change negotiations. However, further (qualitative) research is needed to assess the direct influence health actors have on climate change decision-making processes and whether increasing health actor’s presence at COP produces more health-responsive climate policies and agreements.

Climate commitments fall short of what is needed, and vested interests within capitalistic structures may continue to foster profit over people’s well-being. While celebrating tentative successes, the health community should continue to emphasise the need to protect the health of current and future generations to ensure climate action matches the magnitude of the threat. This should encompass continued strategic engagement with COP processes, a broader focus on health that extends beyond healthcare and promoting climate action alongside health cobenefits. Without fundamental social change, without phasing out fossil fuels, and without climate justice, the health narrative for climate change cannot bring what it promises: health for all.

## Data Availability

Data are available in a public, open access repository. Link to repository: https://earth.bsc.es/gitlab/ghr/health-community-unfccc-cop
