# Supplementary material for: The global health community at international climate change negotiations
Source: BMJ Glob Health. 2024 Apr 18;9(4):e015292. doi: 10.1136/bmjgh-2024-015292 (PMC11029429; doi:10.1136/bmjgh-2024-015292)

Supplementary materials to "The global health community at international climate change negotiations"

Kim R. van Daalen, *PhD*[1,2,3] Nanine Wyma *MBChB*,[4,5] Johanna Schauer-Berg *MD*,[6] Iris M. Blom *MD*,[7] Juliette Mattijsen *MD*,[8,9] Razan Othman *MBBS*,[10] Mohamed Eissa *MD*,[11] Robbie M. Parks *PhD*,[12] Arthur Wyns *MSc*,[13] Ahmed T. Aboushady *MD*,[14] Muha Hassan, *MBChB,*[15] Tarek Ezzine *BMSc,*[16] Salman Khan *MBBS*,[17,18] Menna-Allah Elsayed Zayed *BSPharm*,[19,21] Sarah Neggazi *PharmD*,[20,22] Lujain Alqodmani *MD*,[23] Rachel Lowe, *PhD*[1,7,24]

Methodology for health community attendance at Conference of the Parties (COPs)

These supplementary materials describe the methodology for assessing the attendance of health actors making up the health community at COP1-COP28 by van Daalen *et al.* (2024).

Study design

The aim of this study was to calculate and analyse the number and proportion of health actors attending Conferences of the Parties (COPs) to the UN Framework Convention on Climate Change (UNFCCC) over time (1995-2023), by COP number, country, type of delegate, United Nations Region, World Bank (WB) income groups, and type of Party to the Convention. These new indicators may support the assessment of the influence of health and health actors in international climate change negotiations and can be compared to the number and proportion of participants from other interest groups, including but not limited to the fossil fuel industry.

Data source and extraction

This analysis focused on the attendance of health actors at the COPs among representatives of Parties to the Convention and Observer States, as well as Observer Organisations. Delegates from the press were excluded from the analysis.

The Parties to the Convention have ratified the Convention and fully engage in negotiations. They predominantly include nation-states, but also include the European Union (EU) and representation from the Catholic Church (i.e., Holy See), former countries (e.g., Yugoslavia, Serbia and Montenegro), and *de jure* sovereign states (i.e., State of Palestine).[1,2]

On the other hand, Observer States are those that have not yet completed their ratification of the Convention, and, therefore, do not yet have the right to vote on decisions.[1,2]

Observer organisations include representatives of i) the United Nations [UN] Secretariat and Related Bodies, ii) UN Specialised Agencies or Related Organisations, iii) Intergovernmental Organisations [IGOs], iv) Non-Governmental Organisations [NGOs] and additionally for COP28 representatives of v) Global Climate Action, vi) Host Country Guests, and vii) Temporary Passes. Observer organisations do not have the right to vote on decisions and have more limited access to the convening (e.g., they do have access to the plenary sessions, but not to smaller Party discussions).[1,2]

NGOs represent a broad spectrum of interests, including representatives from business and industry, environmental groups, indigenous populations, local governments and municipal authorities, research and academic institutes, farming and agriculture, labour unions, women and gender groups, youth groups and health representatives.[1,2]

The official lists of delegates for each event were obtained from the UNFCCC website (https://unfccc.int/documents), from COP1 (1995) to COP28 (2023). For each COP, the UNFCCC typically publishes a provisional list of participants before/during the event and a final list of participants once the COP has concluded. The results of this study are based on the final list (and, where relevant, a corrected list) of participants. A full list of the documentation used can be found in **Supplementary Table 1**.

Following a standardised definition of 'health community' or 'health actor' (see following section on definition) and a standardised extraction protocol, informed by expert knowledge of the co-authors, data on health actors was manually extracted by 13 co-authors, collecting information on:

1. #COP.
2. Full name of the delegate. Referred to as "**Name**" within this document.
3. Type of delegate (i.e., Party, Observer State, UN Secretariat & Related Body, UN Specialised Agency or Related Organisation, IGO, NGO). Referred to as "**Type**" within this document.
4. Nominator of the delegate (i.e., the country or organisation delegation that the person is a part of). Referred to as "**Subtype**" within this document.
5. Function or affiliation of the delegate (e.g., "*Monitoring & Evaluation Officer, Department of Environment, Ministry of Health, Wellness and the Environment*"). Referred to as "**Function**" within this document.

One researcher extracted the initial data, which was then unblinded double-checked by a second researcher (i.e., the second researcher could see the work of the first researcher). This verification process involved checking for various errors, such as incorrect person extraction, missing persons, or duplicate entries. Any discrepancies were resolved through a third arbitrator. To enable the calculation of the percentage of health community attendees of total participation, the total (sum) participation of

each type of delegate was extracted from the summary tables of each COP participant list (**Supplementary Table 1)**. However, the UNFCCC participant lists for COP2 and COP5 do not disaggregate the Parties & Observer States and Observer Organisations further. Therefore, for these COPs, we only have total participant data on these two subgroups (**Supplementary Table 4**).

Health community definition

The health community is made up of health actors. Any information described in the **Type**, **Subtype** and **Function,** was used to identify health actors. To avoid bias and ensure reproducibility, no individual was identified as a health actor based on prior knowledge alone. We refrained from conducting online searches using participant names, as this may have introduced errors and/or bias towards those more easily findable online and those from high-income / Global North countries. See more in *limitations*.

Health actors were broadly defined as any person who provides healthcare or a range of preventive, diagnostic, therapeutic, and technical services to improve human health and wellbeing (e.g., medical doctors, nurses, radiologists, pharmacists), works for an organisation primarily focused on human health (e.g., World Health Organization, Ministries of Health, Bill & Melinda Gates Foundation, Malaria No More), works for an organisation representing patients or people with disabilities (e.g., Sustain Our Abilities), or conducts human health research (e.g., epidemiologists at universities or other research institutions). This includes people in the course of study related to the improvement of human health and wellbeing. We also included people with health functions within organisations not primarily focused on health (e.g., "*Health and Safety Officer in Unifor*"), as well as people with a function not primarily focused on health within health organisations (e.g., Assistant to the Minister of Health, Ministry of Health).

The UNFCCC provides documents in different UN languages (English, Spanish, French, Russian, Chinese, Arabic). English participant lists were used to extract information on health actors. However, as the **Type**, **Subtype** and **Function** within the English documents were sometimes in French and Spanish - lists of helpful English, French and Spanish terms were generated to support the identification of health actors (see **Box 1**). Identification was not limited to these terms.

**Box 1 List of suggested search terms to identify health actors.**
**English terms**: anaesthes* OR androlog* OR anesthes* OR biomed* OR brain OR cardio* OR communicable OR dermatolog* OR disease* OR disab* OR endocrinolog* OR epidemiolog* OR genetic* OR genom* OR geriatric* OR gynecolog* OR infectious OR health OR heart OR hygie* OR hematolog* OR immunolog* OR medic* OR midwife OR neonat* OR nephrolog* OR neurolog* OR neuroscien* OR nurs* OR lung OR obstetric* OR oncolog* OR ophthamolog* OR ortho* OR

paediatric* OR patholog* OR paramedic OR pediatric* OR pharmac* OR psychiatr* OR psych* OR physi* OR pulmon* OR radiolog* OR rheumatolog* OR surger* OR surgical OR urolog* OR MD.
**French terms:** anesthés* OR androlog* OR biomédic* OR cerveau OR chirurg* OR cardio* OR cœur OR dermatolog* OR endocrin* OR épidémiolog* OR génétique OR génom* OR gériatri* gynécol* OR handicap* OR hématol* OR hygièn* OR infirmier* OR infectieu* OR invalidit* OR immunol* OR néonatal* OR néphrol* OR neuro OR malad* OR obstétric* OR oncol* OR opthalmol* OR ortho OR médic* OR paramédic* OR pathol* OR pédiatr* OR pharma OR psych* OR poumon OR pulmon* OR radiolog* OR rhumatol* OR santé OR sage femme OR sage-femme OR transmis* OR urolog* OR MD
**Spanish terms:** anestes* OR OR anestés* OR androl* OR biomédico* OR cerebro OR cardio* OR cirug* OR cirujan* OR communicable* OR dermato* OR discapacidad* OR enfermedad* OR enferm* OR endocrin* OR epidemiol* OR farma* OR genétic* OR genóm* OR geriátric* OR ginecol* OR invalid* OR infectious* OR higien* OR hermatól* OR inmunolog* OR neonatol* OR nefrol* OR neuro medic* OR médic OR pulmón OR obstétric* OR oncol* OR oftalmol* OR ortol* OR pediátric* OR patolog* OR paramédic OR partera OR pediátric OR psiquiat* OR psicol* OR radiolog* OR reumatol* OR urólog* OR salud OR sanidad

When it was not immediately clear from the name of the organisation (in **Type**, **Subtype** or **Function**) whether an organisation was a health organisation or not (e.g., CBM UK, Medact, Margaret Pyke Trust, Karuna Foundation, NHS England, Wellcome Trust), a Google Search of the organisation was performed. Note that, as the term "doctor" is used to refer both to i) medical doctors and ii) doctors of philosophy (PhDs), this term on its own was not used to identify someone as a health actor. MD was used to identify health actor where it was used as an abbreviation for medical doctor, but not when used as a common prefix for "Mohammod", "Muhammod", "Muhammed" or "Muhammad" (Md).

Data cleaning and coding

Countries or groups of countries that underwent name changes in the last 28 years (but did not change geographic boundaries) were re-coded to their current (2024) country name (e.g. Swaziland to Eswatini, Zaire to Democratic Republic of the Congo, Turkey to Türkiye, European Community to European Union). Countries that changed geographic boundaries and/or geopolitical context were not re-coded (e.g. Yugoslavia, Serbia and Montenegro) [see **Supplementary Table 2**].

Each Party was assigned to their corresponding (UN) country region (Asia-Pacific States, Eastern European States, African States, Western European and other States, Latin American and Caribbean States), WB income grouping 2022 (high-income, upper-middle-income, lower-middle-income, low-

income), and type of Party to the Convention (i.e., Annex I, and Non-Annex I Parties) [see **Supplementary Table 3**].

To be able to include (former) countries in longitudinal analyses, they have been grouped following their UN region groupings that they would theoretically fall in based on their geographic location (e.g. Yugoslavia was categorised under "Eastern Europe"). These categorisations can be found in **Supplementary Table 3**, and are marked in light blue.

Annex I Parties include the industrialised countries that were members of the Organisation for Economic Co-operation and Development (OECD) in 1992, plus the countries with economies in transition (EIT), including the Russian Federation, the Baltic States, and several Central and Eastern European States. Non-Annex I Parties are mostly 'developing countries', recognised by the Convention as being "especially vulnerable to the adverse impacts of climate change, including countries with low-lying coastal areas and those prone to desertification and drought". Note, whilst the authors view the term 'developing countries' as outdated, it has been used here to align with UNFCCC terminology.

Two categorical variables were manually created to track the attendance of i) Ministers of Health and ii) representatives of Ministries of Health. A list with names of Ministries of Health worldwide generated by the Geneva Foundation of Medical Education and Research (GFMER) was used as a reference list (https://www.gfmer.ch/000_Homepage_En.htm). Ministers or Ministries that combined health and environmental responsibilities (e.g., the Ministry of Health, Wellness and Environment of Antigua and Barbuda, Santé publique, Sécurité de la Chaîne alimentaire et Environnement of Belgium, and the Ministry of Health, Wellness and Environment of Saint Vincent and the Grenadines) were separately tracked (i.e., representatives of the Ministry of Health = 1, Ministry of Health and Environment = 2 , no Ministry of Health = 0). Considering that Ministries of Health may undergo name changes when the national political landscape changes, a Google search of the **Function** description was used to double-check whether the person was affiliated with a Ministry of Health in case of doubt. One further categorical variable (i.e., yes = 1, no = 0) was manually created to track participants included in the health actor definition, who work for organisations involved in fundamentally health-harming practices such as the exploration, production, refining, distribution, marketing or import/export of oil, coal, petroleum, (natural) gas or biomass (e.g., Kuwait Integrated Petroleum Industries Company, British Petroleum Company, Abu Dhabi National Oil Company). A second researcher double-checked all manually created variables before analysis, and a third arbitrator settled disagreements.

Data analysis and visualisation

Simple descriptive statistics were generated to highlight the absolute and proportional participation of health actors over time across different groupings. All analyses and visualisations were generated in R version 4.0.5 (R Foundation, Vienna, Austria, www.r-project.org). For data visualisation, the tidyverse, dplyr, metbrewer, and ggplot packages were used.

Data accessibility

Data and code to reproduce all analyses is publicly available at Gitlab via: https://earth.bsc.es/gitlab/ghr/health-community-unfccc-cop.

Ethical Considerations

All data used for this study was publicly available and accessible, eliminating the need for additional ethical approval.

Research team

The research team comprised an internationally diverse group of early-career researchers from a wide variety of socio-cultural backgrounds (Algeria, Austria, Belgium, Egypt, Finland, India, the Netherlands, Somaliland, South Africa, Spain, Sudan, Tunisia, United Kingdom, United States) and languages (Arabic, Afrikaans, Dutch, English, Finnish, French, German, Hindi, Somali, Spanish) which offered a relevant diversity of perspectives and insights. The team includes a range of health community representatives including biomedical scientists, biostatisticians, epidemiologists, medical doctors, pharmacists, policy advisors, and those in training for these professions (i.e., students).

Limitations

Firstly, not all health actors may have been affiliated with a health organisation or included a health-related descriptor in their function. Therefore, by not performing a manual online search of each individual COP participant (note, >400,000 participants), it is likely that the data presented here is an underestimation of the true number of health actors that attended COP1-28. However, this likely applies to all types of participants and therefore would not change relative counts between types of participants. Secondly, by including all individuals working in health organisations (such as Ministries of Health), individuals with a non-health function within a health organisation have also been included. This may

have led to a possible overestimation of the true number of health actors. Given the weight of the former limitation, it is more likely that overall, this data underestimated the "*true attendance*" of health actors at COP1-COP28. Thirdly, our analyses relied on summary tables in the COP participant lists to acquire the total number of participants. Whilst these total values were provided by delegation type (i.e., Party, Observer State, UN Secretariat & Related Body, UN Specialised Agency or Related Organisations, IGO and NGO), total participant sums were not provided by United Nations Region, World Bank (WB) income groups, and type of Party to the Convention. This limited our ability to explore the health actor data beyond absolute numbers for these subgroupings (e.g., calculating percentages/proportions of health actors in each UN regional grouping). Furthermore, it was deemed inappropriate to use population size as a proxy for the sum of COP participants per subgrouping, as population size does not directly translate to COP delegation size.[1]

References

1. McSweeney, R. Analysis: How delegations at COP climate summits have changed over time. https://www.carbonbrief.org/analysis-how-delegations-at-cop-climate-summits-have-changed-over-time/.
2. What are Parties & non-Party stakeholders? https://unfccc.int/process-and-meetings/what-are-parties-non-party-stakeholders.

Supplementary Table 1. Overview with documentation used to extract delegation members for each Conference of the Parties (1-28).

COP	Location	Date(s)	Publication date	Documentation reference	Link to source
1	Berlin, Germany	28/03-07/04 1995	06/04/1995	FCCC/1995/INF.5/Rev.2	https://ccsr.aori.u-tokyo.ac.jp/old/unfccc3/records/600000324.html
2	Geneva, Switzerland	08/07-19/07 1996	19/07/1996	FCCC/CP/1996/INF.2	https://unfccc.int/cop3/fccc/listpart/particip.html
3	Kyoto, Japan	1/12-10/12 1997	09/12/1997	FCCC/CP/1997/INF.5	https://digitallibrary.un.org/record/690376?ln=en
4	Buenos Aires, Argentina	02/11-13/11 1998	12/11/1998	FCCC/CP/1998/INF.8	https://ccsr.aori.u-tokyo.ac.jp/old/unfccc1/records/600000798.html
5	Bonn, Germany	25/10-5/11 1999	4/11/1999	FCCC/CP/1999/INF.3	https://unfccc.int/cop5/resource/docs99.html
6	Bonn, Germany	16/07-27/07 2001	26/07/2001	FCCC/CP/2001/INF.2	https://ccsr.aori.u-tokyo.ac.jp/old/unfccc3/records/600001050.html
7	Marrakesh, Morroco	29/10-9/11 2001	08/11/2001	FCCC/CP/2001/INF.4	https://ccsr.aori.u-tokyo.ac.jp/old/unfccc3/records/600001707.html
8	New Delhi, India	23/10-01/11 2002	1/11/2002	FCCC/CP/2002/INF.2	https://ccsr.aori.u-tokyo.ac.jp/old/unfccc3/records/600002189.html
9	Milan, Italy	01/12-12/12 2003	11/12/2003	FCCC/CP/2003/INF.1	https://unfccc.int/documents/3541
10	Buenos Aires, Argentina	06/12-17/12 2004	17/12/2004	FCCC/CP/2004/INF.3	https://unfccc.int/resource/docs/cop10/inf03.pdf
11	Montreal, Canada	28/11-09/12 2005	9/12/2005	FCCC/CP/2005/INF.2 (Part 1)<br>FCCC/CP/2005/INF.2 (Part 2)	https://unfccc.int/resource/docs/2005/cop11/eng/inf02p01.pdf<br>https://unfccc.int/resource/docs/2005/cop11/eng/inf02p02.pdf
12	Nairobi, Kenya	06/11-17/11 2006	16/12/2006	FCCC/CP/2006/INF.1	https://unfccc.int/resource/docs/2006/cop12/eng/inf01.pdf
13	Bali, Indonesia	03/12-14/12 2007	14/12/2007	FCCC/CP/2007/INF.1 (Part 1)<br>FCCC/CP/2007/INF.1 (Part 2)	https://unfccc.int/documents/5006<br>https://unfccc.int/documents/5007
14	Poznan, Poland	01/12-12/12 2008	11/12/2008	FCCC/CP/2008/INF.1 (Part 1)<br>FCCC/CP/2008/INF.1 (Part 2)	https://unfccc.int/documents/5459<br>https://unfccc.int/documents/5460

15	Copenhagen, Denmark	07/12-18/12 2009	16/03/2010	FCCC/CP/2009/INF.1(Part 1)<br>FCCC/CP/2009/INF.1(Part 2)<br>FCCC/CP/2009/INF.1(Part 3)	https://unfccc.int/documents/6107<br>https://unfccc.int/documents/6108<br>https://unfccc.int/documents/6109
16	Cancun, Mexico	29/11-10/12 2010	10/12/2010	FCCC/CP/2010/INF.1 (Part 1)<br>FCCC/CP/2010/INF.1 (Part 2)<br>FCCC/CP/2010/INF.1 (Part 3)	https://unfccc.int/documents/6498<br>https://unfccc.int/documents/6499<br>https://unfccc.int/documents/6500
17	Durban, South Africa	28/11-9/12 2011	08/12/2011	FCCC/CP/2011/INF.3 (Part 1)<br>FCCC/CP/2011/INF.3 (Part 2)<br>FCCC/CP/2011/INF.3 (Part 3)	https://unfccc.int/documents/6989<br>https://unfccc.int/documents/6990<br>https://unfccc.int/documents/6991
18	Doha, Qatar	26/11-7/12 2012	07/12/2012	FCCC/CP/2012/INF.2	https://unfccc.int/documents/7631
19	Warsaw, Poland	11/11-22/11 2013	21/11/2013	FCCC/CP/2013/INF.4	https://unfccc.int/documents/8063
20	Lima, Peru	01/12-12/12 2014	12/12/2014	FCCC/CP/2014/INF.2	https://unfccc.int/documents/8579
21	Paris, France	30/11-11/12 2015	11/12/2015	FCCC/CP/2015/INF/2 (Part 1)<br>FCCC/CP/2015/INF.3 (Part 2)<br>FCCC/CP/2015/INF.3 (Part 3)	https://unfccc.int/documents/8984<br>https://unfccc.int/documents/9059<br>https://unfccc.int/documents/9058
22	Marrakech, Morocco	07/11-18/11 2016	18/11/2016	FCCC/CP/2016/INF.3 (Part 1)<br>FCCC/CP/2016/INF.3 (Part 2)<br>FCCC/CP/2016/INF.3 (Part 3)	https://unfccc.int/documents/9494<br>https://unfccc.int/documents/9612<br>https://unfccc.int/documents/9613
23	Bonn, Germany	07/11-17/11 2017	17/11/2017	FCCC/CP/2017/INF.4	https://unfccc.int/documents/28363
24	Katowice, Poland	02/12-14/12 2018	14/12/2018	FCCC/CP/2018/INF.3	https://unfccc.int/documents/187488
25	Madrid, Spain	02/12-13/12 2019	13/12/2019	FCCC/CP/2019/INF.4	https://unfccc.int/documents/184482
26	Glasgow, UK	31/10-12/11 2021	23/11/2021	FCCC/CP/2021/INF.3 (Part 1)<br>FCCC/CP/2021/INF.3 (Part 2)	https://unfccc.int/sites/default/files/resource/cp2021_inf03p01.pdf<br>https://unfccc.int/sites/default/files/resource/cp2021_inf03p02.pdf

27	Sharm el-Sheikh, Egypt	06/11-18/11 2022	02/12/2022	FCCC/CP/2022/INF.3 (Part 1)<br>FCCC/CP/2022/INF.3 (Part 2)	https://unfccc.int/documents/624508<br>https://unfccc.int/documents/624509
28	Dubai, United Arab Emirates	30/11-13/12 2023	15/01/2024<br>22/12/2023	FCCC/CP/2023/INF.3/Rev.1<br>FCCC/CP/2023/INF.3<br>(on-site & virtual-only participation)	https://unfccc.int/documents/636761<br>https://unfccc.int/documents/636675<br>https://unfccc.int/documents/636674

Supplementary Table 2. Recoding of (former) countries and their geopolitical context.

**Recoded (former country names)**		
**Extracted as**	**Recoded as**	**Geopolitical context**
Swaziland	Eswatini	Swaziland was the English name for the Kingdom of Eswatini. Swaziland was a British protectorate from 1903-1968. The name was officially changed to Eswatini (the Swazi language) in 2018.
Zaire	Democratic Republic of the Congo	Following a coup in 1965, the country was renamed as the Republic of Zaire in 1971 until 1997 when its name reverted to the Democratic Republic of the Congo.
Libyan Arab Jamahirya	Libya	The fall of the last pro-Gaddafi site and Gaddafi's assassination in 2011 marked the end of the Libyan Arab Jamahirya.
Former Yugoslav Republic of Macedonia	Republic of North Macedonia	In 1991, the Republic of Macedonia (which was renamed as the Republic of North Macedonia in 2019) became one of the successor states of Yugoslavia.
Turkey	Türkiye	Following a formal request of the Turkish authorities Turkey has been officially recognised as Türkiye in international organisations since 2022.
European Community	European Union	The European Community (EC) was an economic association formed by six European member countries in 1957, consisting of three communities that eventually were replaced by the European Union (EU) in 1993.
**Not recoded, including (former) countries**		
Holy See	The Holy See is the universal government of the Catholic Church. It operates from the Vatican City State which is a sovereign, independent territory. The Holy See has a permanent observer status at the United Nations.	
Palestine	Palestine is considered to be a de jure sovereign state in Western Asia, comprising the Gaza strip, West Bank and parts of modern Israel.	
Serbia and Montenegro	Serbia and Montenegro existed between 1992-2006, when it was dissolved after the breakup of Yugoslavia.	
Yugoslavia	A nation that was founded after WWI and was dissolved after a number of conflicts in the early 1990s. Was made up of what is present day Bosnia and Herzegovina, Croatia, North Macedonia, Montenegro, Serbia (including the regions of Kosovo and Vojvodina) and Slovenia.	

Supplementary Table 3. Party and Observer State groupings by commitment to the Convention, UN regional grouping and WB income grouping. To be able to include (former) countries in longitudinal analyses, they have been grouped following their UN region groupings that they would theoretically fall in based on their geographic location (e.g. Yugoslavia was categorised under "Eastern Europe"). These categorisations based on geography are marked in light blue.

Country	Commitment to Convention	UN regional grouping	WB income grouping
Afghanistan	Non-Annex 1	Asia-Pacific States	Low-income
Albania	Non-Annex 1	Eastern European States	Upper-middle income
Algeria	Non-Annex 1	African States	Lower-middle income
Andorra	Non-Annex 1	Western European and other States	High-income
Angola	Non-Annex 1	African States	Lower-middle income
Antigua and Barbuda	Non-Annex 1	Latin American and Caribbean States	High-income
Argentina	Non-Annex 1	Latin American and Caribbean States	Upper-middle income
Armenia	Non-Annex 1	Eastern European States	Upper-middle income
Australia	Annex 1	Western European and other States	High-income
Austria	Annex 1	Western European and other States	High-income
Azerbaijan	Non-Annex 1	Eastern European States	Upper-middle income
Bahamas	Non-Annex 1	Latin American and Caribbean States	High-income
Bahrain	Non-Annex 1	Asia-Pacific States	High-income
Bangladesh	Non-Annex 1	Asia-Pacific States	Lower-middle income

Barbados	Non-Annex 1	Latin American and Caribbean States	High-income
Belarus	Annex 1	Eastern European States	Upper-middle income
Belgium	Annex 1	Western European and other States	High-income
Belize	Non-Annex 1	Latin American and Caribbean States	Lower-middle income
Benin	Non-Annex 1	African States	Lower-middle income
Bhutan	Non-Annex 1	Asia-Pacific States	Lower-middle income
Bolivia	Non-Annex 1	Latin American and Caribbean States	Lower-middle income
Bosnia and Herzegovina	Non-Annex 1	Eastern European States	Upper-middle income
Botswana	Non-Annex 1	African States	Upper-middle income
Brazil	Non-Annex 1	Latin American and Caribbean States	Upper-middle income
Brunei Darussalam	Non-Annex 1	Asia-Pacific States	High-income
Bulgaria	Annex 1	Eastern European States	Upper-middle income
Burkina Faso	Non-Annex 1	African States	Low-income
Burundi	Non-Annex 1	African States	Low-income
Cambodia	Non-Annex 1	Asia-Pacific States	Lower-middle income
Cameroon	Non-Annex 1	African States	Lower-middle income
Canada	Annex 1	Western European and other States	High-income
Cape Verde	Non-Annex 1	African States	Lower-middle income
Central African Republic	Non-Annex 1	African States	Low-income
Chad	Non-Annex 1	African States	Low-income

Chile	Non-Annex 1	Latin American and Caribbean States	High-income
China	Non-Annex 1	Asia-Pacific States	Upper-middle income
Colombia	Non-Annex 1	Latin American and Caribbean States	Upper-middle income
Comoros	Non-Annex 1	African States	Lower-middle income
Cook Islands	Non-Annex 1	Asia-Pacific States	No classification
Costa Rica	Non-Annex 1	Latin American and Caribbean States	Upper-middle income
Côte d'Ivoire	Non-Annex 1	African States	Lower-middle income
Croatia	Annex 1	Eastern European States	High-income
Cuba	Non-Annex 1	Latin American and Caribbean States	Upper-middle income
Cyprus	Annex 1	Asia-Pacific States	High-income
Czech Republic	Annex 1	Eastern European States	High-income
Democratic Republic of the Congo	Non-Annex 1	African States	Low-income
Denmark	Annex 1	Western European and other States	High-income
Djibouti	Non-Annex 1	African States	Lower-middle income
Dominica	Non-Annex 1	Latin American and Caribbean States	Upper-middle income
Dominican Republic	Non-Annex 1	Latin American and Caribbean States	Upper-middle income
Ecuador	Non-Annex 1	Latin American and Caribbean States	Upper-middle income
Egypt	Non-Annex 1	African States	Lower-middle income
El Salvador	Non-Annex 1	Latin American and Caribbean States	Lower-middle income

Equatorial Guinea	Non-Annex 1	African States	Upper-middle income
Eritrea	Non-Annex 1	African States	Low-income
Estonia	Annex 1	Eastern European States	High-income
Eswatini	Non-Annex 1	African States	Lower-middle income
Ethiopia	Non-Annex 1	African States	Low-income
European Union	Annex 1	Western European and other States	No classification
Fiji	Non-Annex 1	Asia-Pacific States	Upper-middle income
Finland	Annex 1	Western European and other States	High-income
France	Annex 1	Western European and other States	High-income
Gabon	Non-Annex 1	African States	Upper-middle income
Gambia	Non-Annex 1	African States	Low-income
Georgia	Non-Annex 1	Eastern European States	Upper-middle income
Germany	Annex 1	Western European and other States	High-income
Ghana	Non-Annex 1	African States	Lower-middle income
Greece	Annex 1	Western European and other States	High-income
Grenada	Non-Annex 1	Latin American and Caribbean States	Upper-middle income
Guatemala	Non-Annex 1	Latin American and Caribbean States	Upper-middle income
Guinea	Non-Annex 1	African States	Low-income
Guinea-Bissau	Non-Annex 1	African States	Low-income
Guyana	Non-Annex 1	Latin American and Caribbean States	Upper-middle income

Haiti	Non-Annex 1	Latin American and Caribbean States	Lower-middle income
Holy See	No classification	Western European and other States	No classification
Honduras	Non-Annex 1	Latin American and Caribbean States	Lower-middle income
Hungary	Annex 1	Eastern European States	High-income
Iceland	Annex 1	Western European and other States	High-income
India	Non-Annex 1	Asia-Pacific States	Lower-middle income
Indonesia	Non-Annex 1	Asia-Pacific States	Lower-middle income
Iran (Islamic Republic of)	Non-Annex 1	Asia-Pacific States	Lower-middle income
Iraq	Non-Annex 1	Asia-Pacific States	Upper-middle income
Ireland	Annex 1	Western European and other States	High-income
Israel	Non-Annex 1	Western European and other States	High-income
Italy	Annex 1	Western European and other States	High-income
Jamaica	Non-Annex 1	Latin American and Caribbean States	Upper-middle income
Japan	Annex 1	Asia-Pacific States	High-income
Jordan	Non-Annex 1	Asia-Pacific States	Upper-middle income
Kazakhstan	Non-Annex 1	Asia-Pacific States	Upper-middle income
Kenya	Non-Annex 1	African States	Lower-middle income
Kiribati	Non-Annex 1	Asia-Pacific States	Lower-middle income
Kuwait	Non-Annex 1	Asia-Pacific States	High-income
Kyrgyzstan	Non-Annex 1	Asia-Pacific States	Lower-middle income

Lao People's Democratic Republic	Non-Annex 1	Asia-Pacific States	Lower-middle income
Latvia	Annex 1	Eastern European States	High-income
Lebanon	Non-Annex 1	Asia-Pacific States	Upper-middle income
Lesotho	Non-Annex 1	African States	Lower-middle income
Liberia	Non-Annex 1	African States	Low-income
Libya	Non-Annex 1	African States	Upper-middle income
Liechtenstein	Annex 1	Western European and other States	High-income
Lithuania	Annex 1	Eastern European States	High-income
Luxembourg	Annex 1	Western European and other States	High-income
Madagascar	Non-Annex 1	African States	Low-income
Malawi	Non-Annex 1	African States	Low-income
Malaysia	Non-Annex 1	Asia-Pacific States	Upper-middle income
Maldives	Non-Annex 1	Asia-Pacific States	Upper-middle income
Mali	Non-Annex 1	African States	Low-income
Malta	Annex 1	Western European and other States	High-income
Marshall Islands	Non-Annex 1	Asia-Pacific States	Upper-middle income
Mauritania	Non-Annex 1	African States	Lower-middle income
Mauritius	Non-Annex 1	African States	Upper-middle income
Mexico	Non-Annex 1	Latin American and Caribbean States	Upper-middle income

Micronesia (Federated States of)	Non-Annex 1	Asia-Pacific States	Lower-middle income
Moldova	Non-Annex 1	Eastern European States	Upper-middle income
Monaco	Annex 1	Western European and other States	High-income
Mongolia	Non-Annex 1	Asia-Pacific States	Lower-middle income
Montenegro	Non-Annex 1	Eastern European States	Upper-middle income
Morocco	Non-Annex 1	African States	Lower-middle income
Mozambique	Non-Annex 1	African States	Low-income
Myanmar	Non-Annex 1	Asia-Pacific States	Lower-middle income
Namibia	Non-Annex 1	African States	Upper-middle income
Nauru	Non-Annex 1	Asia-Pacific States	High-income
Nepal	Non-Annex 1	Asia-Pacific States	Lower-middle income
Netherlands	Annex 1	Western European and other States	High-income
New Zealand	Annex 1	Western European and other States	High-income
Nicaragua	Non-Annex 1	Latin American and Caribbean States	Lower-middle income
Niger	Non-Annex 1	African States	Low-income
Nigeria	Non-Annex 1	African States	Lower-middle income
Niue	Non-Annex 1	Asia-Pacific States	No classification
North Korea	Non-Annex 1	Asia-Pacific States	Low-income
North Macedonia	Non-Annex 1	Eastern European States	Upper-middle income

Norway	Annex 1	Western European and other States	High-income
Oman	Non-Annex 1	Asia-Pacific States	High-income
Pakistan	Non-Annex 1	Asia-Pacific States	Lower-middle income
Palau	Non-Annex 1	Asia-Pacific States	High-income
Palestine	No classification	Western European and other States	No classification
Panama	Non-Annex 1	Latin American and Caribbean States	Upper-middle income
Papua New Guinea	Non-Annex 1	Asia-Pacific States	Lower-middle income
Paraguay	Non-Annex 1	Latin American and Caribbean States	Upper-middle income
Peru	Non-Annex 1	Latin American and Caribbean States	Upper-middle income
Philippines	Non-Annex 1	Asia-Pacific States	Lower-middle income
Poland	Annex 1	Eastern European States	High-income
Portugal	Annex 1	Western European and other States	High-income
Qatar	Non-Annex 1	Asia-Pacific States	High-income
Republic of Moldova	Non-Annex 1	Eastern European States	Upper-middle income
Republic of the Congo	Non-Annex 1	African States	Lower-middle income
Romania	Annex 1	Eastern European States	Upper-middle income
Russian Federation	Annex 1	Eastern European States	Upper-middle income
Rwanda	Non-Annex 1	African States	Low-income
Saint Kitts and Nevis	Non-Annex 1	Latin American and Caribbean States	High-income
Saint Lucia	Non-Annex 1	Latin American and Caribbean States	Upper-middle income

Saint Vincent and the Grenadines	Non-Annex 1	Latin American and Caribbean States	Upper-middle income
Samoa	Non-Annex 1	Asia-Pacific States	Lower-middle income
San Marino	Non-Annex 1	Western European and other States	High-income
Sao Tome and Principe	Non-Annex 1	African States	Lower-middle income
Saudi Arabia	Non-Annex 1	Asia-Pacific States	High-income
Senegal	Non-Annex 1	African States	Lower-middle income
Serbia	Non-Annex 1	Eastern European States	Upper-middle income
Serbia and Montenegro	No classification	Eastern European States	No classification
Seychelles	Non-Annex 1	African States	High-income
Sierra Leone	Non-Annex 1	African States	Low-income
Singapore	Non-Annex 1	Asia-Pacific States	High-income
Slovakia	Annex 1	Eastern European States	High-income
Slovenia	Annex 1	Eastern European States	High-income
Solomon Islands	Non-Annex 1	Asia-Pacific States	Lower-middle income
Somalia	Non-Annex 1	African States	Low-income
South Africa	Non-Annex 1	African States	Upper-middle income
South Korea	Non-Annex 1	Asia-Pacific States	High-income
South Sudan	Non-Annex 1	African States	Low-income
Spain	Annex 1	Western European and other States	High-income

Sri Lanka	Non-Annex 1	Asia-Pacific States	Lower-middle income
Sudan	Non-Annex 1	African States	Low-income
Suriname	Non-Annex 1	Latin American and Caribbean States	Upper-middle income
Sweden	Annex 1	Western European and other States	High-income
Switzerland	Annex 1	Western European and other States	High-income
Syrian Arab Republic	Non-Annex 1	Asia-Pacific States	Low-income
Tajikistan	Non-Annex 1	Asia-Pacific States	Lower-middle income
Thailand	Non-Annex 1	Asia-Pacific States	Upper-middle income
Timor-Leste	Non-Annex 1	Asia-Pacific States	Lower-middle income
Togo	Non-Annex 1	African States	Low-income
Tonga	Non-Annex 1	Asia-Pacific States	Upper-middle income
Trinidad and Tobago	Non-Annex 1	Latin American and Caribbean States	High-income
Tunisia	Non-Annex 1	African States	Lower-middle income
Türkiye	Annex 1	Asia-Pacific States	Upper-middle income
Turkmenistan	Non-Annex 1	Asia-Pacific States	Upper-middle income
Tuvalu	Non-Annex 1	Asia-Pacific States	Upper-middle income
Uganda	Non-Annex 1	African States	Low-income
Ukraine	Annex 1	Eastern European States	Lower-middle income
United Arab Emirates	Non-Annex 1	Asia-Pacific States	High-income
United Kingdom of Great	Annex 1	Western European and other States	

Britain and Northern Ireland			
United Republic of Tanzania	Non-Annex 1	African States	Lower-middle income
United States of America	Annex 1	Western European and other States	High-income
Uruguay	Non-Annex 1	Latin American and Caribbean States	High-income
Uzbekistan	Non-Annex 1	Asia-Pacific States	Lower-middle income
Vanuatu	Non-Annex 1	Asia-Pacific States	Lower-middle income
Venezuela (Bolivarian Republic of)	Non-Annex 1	Latin American and Caribbean States	No classification
Viet Nam	Non-Annex 1	Asia-Pacific States	Lower-middle income
Yemen	Non-Annex 1	Asia-Pacific States	Low-income
Yugoslavia	No classification	Eastern European States	No classification
Zambia	Non-Annex 1	African States	Lower-middle income
Zimbabwe	Non-Annex 1	African States	Lower-middle income

Supplementary Table 4. Absolute number and percentage of health actors' attendance by delegate type and COP over time (1995-2023). Overall sums of delegates from parties and observers were taken from the summary tables at the top of the participant lists used for this study. However, the UNFCCC participant lists for COP2 and COP5 do not disaggregate the Parties & Observer States and Observer Organisations further.

COP	Type	Health	TotalPax	%	City	Country
1	Party	8	757	1.1	Berlin	Germany
1	Observer State	6	112	5.4	Berlin	Germany
1	UN Secretariat or Related Body	0	26	0.0	Berlin	Germany
1	Specialised Agency or Related Organisation	0	28	0.0	Berlin	Germany
1	IGO	0	23	0.0	Berlin	Germany
1	NGO	8	979	0.8	Berlin	Germany
1	**Parties & Observer States**	**14**	**869**	**1.6**	Berlin	Germany
1	**Observer Organisations**	**8**	**1056**	**0.8**	Berlin	Germany
1	**Total**	**22**	**1925**	**1.1**	Berlin	Germany
2	**Parties & Observer States**	**2**	**970**	**0.2**	Geneva	Switzerland
2	**Observer Organisations**	**15**	**614**	**2.4**	Geneva	Switzerland
2	**Total**	**17**	**1584**	**1.1**	Geneva	Switzerland
3	Party	9	1534	0.6	Kyoto	Japan
3	Observer State	1	29	3.4	Kyoto	Japan
3	UN Secretariat or Related Body	0	90	0.0	Kyoto	Japan
3	Specialised Agency or Related Organisation	3	33	9.1	Kyoto	Japan
3	IGO	0	79	0.0	Kyoto	Japan
3	NGO	64	3663	1.7	Kyoto	Japan
3	**Parties & Observer States**	**10**	**2273**	**0.4**	Kyoto	Japan
3	**Observer Organisations**	**67**	**3865**	**1.7**	Kyoto	Japan
3	**Total**	**77**	**6138**	**1.3**	Kyoto	Japan
4	Party	2	1391	0.1	Buenos Aires	Argentina
4	Observer State	0	39	0.0	Buenos Aires	Argentina
4	UN Secretariat or Related Body	0	68	0.0	Buenos Aires	Argentina

van Daalen KR, *et al. BMJ Glob Health* 2024; 9:e015292. doi: 10.1136/bmjgh-2024-015292

4	Specialised Agency or Related Organisation	5	83	6.0	Buenos Aires	Argentina
4	IGO	0	120	0.0	Buenos Aires	Argentina
4	NGO	15	2357	0.6	Buenos Aires	Argentina
4	**Parties & Observer States**	**2**	**1430**	0.1	Buenos Aires	Argentina
4	**Observer Organisations**	**20**	**2628**	0.8	Buenos Aires	Argentina
4	**Total**	**22**	**4058**	0.5	Buenos Aires	Argentina
5	**Parties & Observer States**	**2**	**1653**	**0.1**	Bonn	Germany
5	**Observer Organisations**	**37**	**2001**	**1.8**	Bonn	Germany
5	**Total**	**39**	**3654**	**1.1**	Bonn	Germany
6	Party	15	1813	0.8	The Hague	Netherlands
6	Observer State	0	6	0.0	The Hague	Netherlands
6	UN Secretariat or Related Body	0	49	0.0	The Hague	Netherlands
6	Specialised Agency or Related Organisation	3	38	7.9	The Hague	Netherlands
6	IGO	0	49	0.0	The Hague	Netherlands
6	NGO	31	1487	2.1	The Hague	Netherlands
6	**Parties & Observer States**	**15**	**1819**	**0.8**	The Hague	Netherlands
6	**Observer Organisations**	**34**	**1723**	**2.0**	The Hague	Netherlands
6	**Total**	**49**	**3542**	**1.4**	The Hague	Netherlands
7	Party	15	2414	0.6	Marrakech	Morocco
7	Observer State	0	18	0.0	Marrakech	Morocco
7	UN Secretariat or Related Body	0	57	0.0	Marrakech	Morocco
7	Specialised Agency or Related Organisation	5	67	7.5	Marrakech	Morocco
7	IGO	0	118	0.0	Marrakech	Morocco
7	NGO	12	1327	0.9	Marrakech	Morocco
7	**Parties & Observer States**	**15**	**2432**	**0.6**	Marrakech	Morocco
7	**Observer Organisations**	**17**	**1569**	**1.1**	Marrakech	Morocco
7	**Total**	**32**	**4001**	**0.8**	Marrakech	Morocco
8	Party	6	1456	0.4	New Dehli	India
8	Observer State	0	12	0.0	New Dehli	India
8	UN Secretariat or Related Body	0	66	0.0	New Dehli	India
8	Specialised Agency or Related Organisation	5	63	7.9	New Dehli	India

van Daalen KR, *et al. BMJ Glob Health* 2024; 9:e015292. doi: 10.1136/bmjgh-2024-015292

8	IGO	5	102	4.9	New Dehli	India
8	NGO	6	1858	0.3	New Dehli	India
8	**Parties & Observer States**	**6**	**1468**	**0.4**	New Dehli	India
8	**Observer Organisations**	**16**	**2089**	**0.8**	New Dehli	India
8	**Total**	**22**	**3557**	**0.6**	New Dehli	India
9	Party	14	1931	0.7	Milan	Italy
9	Observer State	0	16	0.0	Milan	Italy
9	UN Secretariat or Related Body	0	72	0.0	Milan	Italy
9	Specialised Agency or Related Organisation	10	95	10.5	Milan	Italy
9	IGO	2	127	1.6	Milan	Italy
9	NGO	30	2404	1.2	Milan	Italy
9	**Parties & Observer States**	**14**	**1947**	**0.7**	Milan	Italy
9	**Observer Organisations**	**42**	**2698**	**1.6**	Milan	Italy
9	**Total**	**56**	**4645**	**1.2**	Milan	Italy
10	Party	9	2210	0.4	Buenos Aires	Argentina
10	Observer State	0	9	0.0	Buenos Aires	Argentina
10	UN Secretariat or Related Body	0	73	0.0	Buenos Aires	Argentina
10	Specialised Agency or Related Organisation	6	90	6.7	Buenos Aires	Argentina
10	IGO	15	96	15.6	Buenos Aires	Argentina
10	NGO	18	2888	0.6	Buenos Aires	Argentina
10	**Parties & Observer States**	**9**	**2219**	**0.4**	Buenos Aires	Argentina
10	**Observer Organisations**	**39**	**3147**	**1.2**	Buenos Aires	Argentina
10	**Total**	**48**	**5366**	**0.9**	Buenos Aires	Argentina
11	Party	24	2804	0.9	Montreal	Canada
11	Observer State	0	5	0.0	Montreal	Canada
11	UN Secretariat or Related Body	0	115	0.0	Montreal	Canada
11	Specialised Agency or Related Organisation	12	119	10.1	Montreal	Canada
11	IGO	1	179	0.6	Montreal	Canada
11	NGO	36	5435	0.7	Montreal	Canada
11	**Parties & Observer States**	**24**	**2809**	0.9	Montreal	Canada
11	**Observer Organisations**	**49**	**5848**	0.8	Montreal	Canada

11	**Total**	**73**	**8657**	0.8	Montreal	Canada
12	Party	16	2344	0.7	Nairobi	Kenya
12	Observer State	0	8	0.0	Nairobi	Kenya
12	UN Secretariat or Related Body	0	141	0.0	Nairobi	Kenya
12	Specialised Agency or Related Organisation	20	98	20.4	Nairobi	Kenya
12	IGO	31	161	19.3	Nairobi	Kenya
12	NGO	5	2533	0.2	Nairobi	Kenya
12	**Parties & Observer States**	**16**	**2352**	**0.7**	Nairobi	Kenya
12	**Observer Organisations**	**56**	**2933**	**1.9**	Nairobi	Kenya
12	**Total**	**72**	**5285**	**1.4**	Nairobi	Kenya
13	Party	32	3508	0.9	Bali	Indonesia
13	Observer State	0	8	0.0	Bali	Indonesia
13	UN Secretariat or Related Body	1	255	0.4	Bali	Indonesia
13	Specialised Agency or Related Organisation	41	251	16.3	Bali	Indonesia
13	IGO	61	316	19.3	Bali	Indonesia
13	NGO	20	4993	0.4	Bali	Indonesia
13	**Parties & Observer States**	**32**	**3516**	**0.9**	Bali	Indonesia
13	**Observer Organisations**	**123**	**5815**	**2.1**	Bali	Indonesia
13	**Total**	**155**	**9331**	**1.7**	Bali	Indonesia
14	Party	29	3958	0.7	Poznań	Poland
14	Observer State	0	9	0.0	Poznań	Poland
14	UN Secretariat or Related Body	2	193	1.0	Poznań	Poland
14	Specialised Agency or Related Organisation	10	152	6.6	Poznań	Poland
14	IGO	30	252	11.9	Poznań	Poland
14	NGO	12	3869	0.3	Poznań	Poland
14	**Parties & Observer States**	**29**	**3967**	**0.7**	Poznań	Poland
14	**Observer Organisations**	**54**	**4463**	**1.2**	Poznań	Poland
14	**Total**	**83**	**8430**	**1.0**	Poznań	Poland
15	Party	87	10583	0.8	Copenhagen	Denmark
15	Observer State	0	15	0.0	Copenhagen	Denmark
15	UN Secretariat or Related Body	7	530	1.3	Copenhagen	Denmark

15	Specialised Agency or Related Organisation	36	336	10.7	Copenhagen	Denmark
15	IGO	37	568	6.5	Copenhagen	Denmark
15	NGO	58	12048	0.5	Copenhagen	Denmark
15	**Parties & Observer States**	**87**	**10591**	**0.8**	Copenhagen	Denmark
15	**Observer Organisations**	**138**	**13482**	**1.0**	Copenhagen	Denmark
15	**Total**	**225**	**24073**	**0.9**	Copenhagen	Denmark
16	Party	53	5183	1.0	Cancún	Mexico
16	Observer State	0	9	0.0	Cancún	Mexico
16	UN Secretariat or Related Body	1	265	0.4	Cancún	Mexico
16	Specialised Agency or Related Organisation	14	210	6.7	Cancún	Mexico
16	IGO	35	351	10.0	Cancún	Mexico
16	NGO	23	4560	0.5	Cancún	Mexico
16	**Parties & Observer States**	**53**	**5192**	**1.0**	Cancún	Mexico
16	**Observer Organisations**	**73**	**5386**	**1.4**	Cancún	Mexico
16	**Total**	**126**	**10578**	**1.2**	Cancún	Mexico
17	Party	41	5399	0.8	Durban	South Africa
17	Observer State	0	14	0.0	Durban	South Africa
17	UN Secretariat or Related Body	4	297	1.3	Durban	South Africa
17	Specialised Agency or Related Organisation	10	258	3.9	Durban	South Africa
17	IGO	35	484	7.2	Durban	South Africa
17	NGO	62	4772	1.3	Durban	South Africa
17	**Parties & Observer States**	**41**	**5413**	**0.8**	Durban	South Africa
17	**Observer Organisations**	**111**	**5811**	**1.9**	Durban	South Africa
17	**Total**	**152**	**11224**	**1.4**	Durban	South Africa
18	Party	28	4343	0.6	Doha	Qatar
18	Observer State	0	13	0.0	Doha	Qatar
18	UN Secretariat or Related Body	0	209	0.0	Doha	Qatar
18	Specialised Agency or Related Organisation	6	135	4.4	Doha	Qatar
18	IGO	37	329	11.2	Doha	Qatar
18	NGO	25	3292	0.8	Doha	Qatar
18	**Parties & Observer States**	**28**	**4356**	**0.6**	Doha	Qatar

18	**Observer Organisations**	**68**	**3965**	**1.7**	Doha	Qatar
18	**Total**	**96**	**8321**	**1.2**	Doha	Qatar
19	Party	34	4011	0.8	Warsaw	Poland
19	Observer State	0	11	0.0	Warsaw	Poland
19	UN Secretariat or Related Body	0	156	0.0	Warsaw	Poland
19	Specialised Agency or Related Organisation	14	145	9.7	Warsaw	Poland
19	IGO	30	363	8.3	Warsaw	Poland
19	NGO	36	3031	1.2	Warsaw	Poland
19	**Parties & Observer States**	**34**	**4022**	**0.8**	Warsaw	Poland
19	**Observer Organisations**	**80**	**3695**	**2.2**	Warsaw	Poland
19	**Total**	**114**	**7717**	**1.5**	Warsaw	Poland
20	Party	27	6291	0.4	Lima	Peru
20	Observer State	0	5	0.0	Lima	Peru
20	UN Secretariat or Related Body	0	245	0.0	Lima	Peru
20	Specialised Agency or Related Organisation	12	197	6.1	Lima	Peru
20	IGO	21	439	4.8	Lima	Peru
20	NGO	28	3104	0.9	Lima	Peru
20	**Parties & Observer States**	**27**	**6296**	**0.4**	Lima	Peru
20	**Observer Organisations**	**61**	**3985**	**1.5**	Lima	Peru
20	**Total**	**88**	**10281**	**0.9**	Lima	Peru
21	Party	104	19208	0.5	Paris	France
21	Observer State	0	52	0.0	Paris	France
21	UN Secretariat or Related Body	0	556	0.0	Paris	France
21	Specialised Agency or Related Organisation	28	415	6.7	Paris	France
21	IGO	69	1037	6.7	Paris	France
21	NGO	95	6306	1.5	Paris	France
21	**Parties & Observer States**	**104**	**19260**	**0.5**	Paris	France
21	**Observer Organisations**	**192**	**8314**	**2.3**	Paris	France
21	**Total**	**296**	**27574**	**1.1**	Paris	France
22	Party	100	15878	0.6	Marrakech	Morocco
22	Observer State	0	7	0.0	Marrakech	Morocco

22	UN Secretariat or Related Body	1	340	0.3	Marrakech	Morocco
22	Specialised Agency or Related Organisation	22	362	6.1	Marrakech	Morocco
22	IGO	20	618	3.2	Marrakech	Morocco
22	NGO	63	4155	1.5	Marrakech	Morocco
22	**Parties & Observer States**	**100**	**15885**	**0.6**	Marrakech	Morocco
22	**Observer Organisations**	**106**	**5475**	**1.9**	Marrakech	Morocco
22	**Total**	**206**	**21360**	**1.0**	Marrakech	Morocco
23	Party	47	9196	0.5	Bonn	Germany
23	Observer State	0	6	0.0	Bonn	Germany
23	UN Secretariat or Related Body	0	465	0.0	Bonn	Germany
23	Specialised Agency or Related Organisation	41	386	10.6	Bonn	Germany
23	IGO	19	597	3.2	Bonn	Germany
23	NGO	65	4095	1.6	Bonn	Germany
23	**Parties & Observer States**	**47**	**9202**	**0.5**	Bonn	Germany
23	**Observer Organisations**	**125**	**5543**	**2.3**	Bonn	Germany
23	**Total**	**172**	**14745**	**1.2**	Bonn	Germany
24	Party	59	11090	0.5	Katowice	Poland
24	Observer State	0	10	0.0	Katowice	Poland
24	UN Secretariat or Related Body	0	216	0.0	Katowice	Poland
24	Specialised Agency or Related Organisation	13	271	4.8	Katowice	Poland
24	IGO	14	652	2.1	Katowice	Poland
24	NGO	65	5054	1.3	Katowice	Poland
24	**Parties & Observer States**	**59**	**11100**	**0.5**	Katowice	Poland
24	**Observer Organisations**	**92**	**6193**	**1.5**	Katowice	Poland
24	**Total**	**151**	**17293**	**0.9**	Katowice	Poland
25	Party	67	11406	0.6	Madrid	Spain
25	Observer State	0	8	0.0	Madrid	Spain
25	UN Secretariat or Related Body	4	306	1.3	Madrid	Spain
25	Specialised Agency or Related Organisation	20	400	5.0	Madrid	Spain
25	IGO	19	652	2.9	Madrid	Spain
25	NGO	85	7417	1.1	Madrid	Spain

van Daalen KR, *et al. BMJ Glob Health* 2024; 9:e015292. doi: 10.1136/bmjgh-2024-015292

25	**Parties & Observer States**	**67**	**11414**	**0.6**	Madrid	Spain
25	**Observer Organisations**	**128**	**8775**	**1.5**	Madrid	Spain
25	**Total**	**195**	**20189**	**1.0**	Madrid	Spain
26	Party	82	9742	0.8	Glasgow	UK
26	Observer State	0	7	0.0	Glasgow	UK
26	UN Secretariat or Related Body	1	361	0.3	Glasgow	UK
26	Specialised Agency or Related Organisation	15	369	4.1	Glasgow	UK
26	IGO	29	741	3.9	Glasgow	UK
26	NGO	243	9529	2.6	Glasgow	UK
26	**Parties & Observer States**	**82**	**9749**	**0.8**	Glasgow	UK
26	**Observer Organisations**	**288**	**11000**	**2.6**	Glasgow	UK
26	**Total**	**370**	**20749**	**1.8**	Glasgow	UK
27	Party	103	11969	0.9	Sharm El-Sheikh	Egypt
27	UN Secretariat or Related Body	0	432	0.0	Sharm El-Sheikh	Egypt
27	Specialised Agency or Related Organisation	21	567	3.7	Sharm El-Sheikh	Egypt
27	IGO	40	1151	3.5	Sharm El-Sheikh	Egypt
27	NGO	227	10090	2.2	Sharm El-Sheikh	Egypt
27	**Parties & Observer States**	**103**	**11969**	**0.9**	Sharm El-Sheikh	Egypt
27	**Observer Organisations**	**288**	**12241**	**2.4**	Sharm El-Sheikh	Egypt
27	**Total**	**391**	**24210**	**1.6**	Sharm El-Sheikh	Egypt
28	Party	436	20579	2.1	Dubai	UAE
28	Party Overflow	273	23771	1.1	Dubai	UAE
28	UN Secretariat or Related Body	5	833	0.6	Dubai	UAE
28	UN Secretariat or Related Body Overflow	3	614	0.5	Dubai	UAE
28	Specialised Agency or Related Organisation	70	822	8.5	Dubai	UAE
28	Specialised Agency or Related Organisation Overflow	0	502	0.0	Dubai	UAE
28	IGO	70	1913	3.7	Dubai	UAE
28	NGO	520	14701	3.5	Dubai	UAE
28	Global Climate Action	4	512	0.8	Dubai	UAE
28	Host country guests	216	4297	5.0	Dubai	UAE
28	Temporary passes	15	805	1.9	Dubai	UAE

28	**Parties & Observer States**	**709**	**44350**	**1.6**	Dubai	UAE
28	**Observer Organisations**	**903**	**24999**	**3.6**	Dubai	UAE
28	**Total**	**1612**	**69349**	**2.3**	Dubai	UAE

Supplementary Figure 1 COP delegates over time (1995-2023). This figure includes representatives of Parties to the Convention and Observer States, as well as Observer Organisations. Delegates from the press were excluded from the analysis.

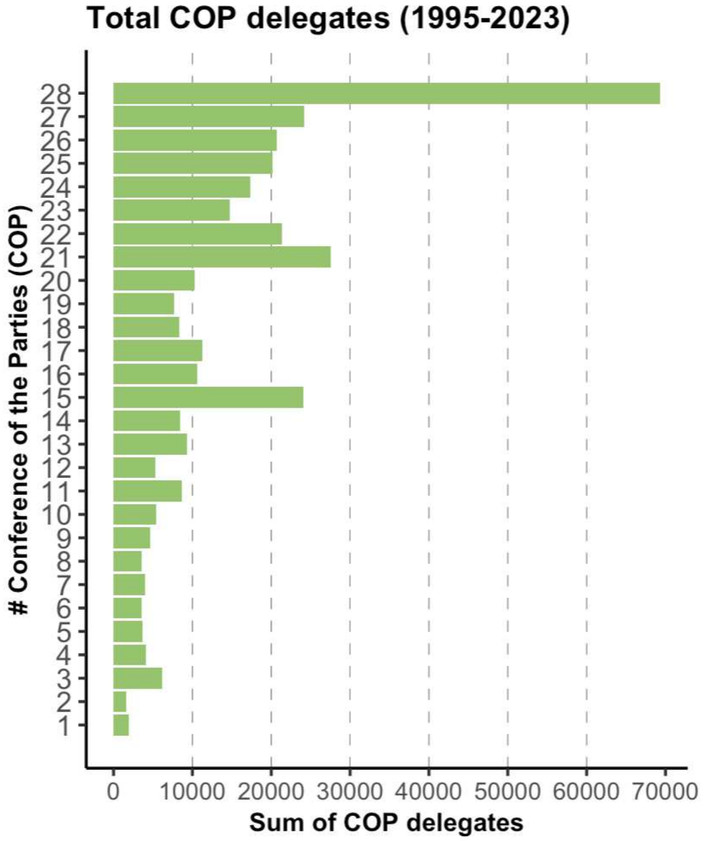

Supplement: Supplementary data [file bmjgh-2024-015292supp001.pdf]
